# Improvement of two Mn^2+^ coordination polymers on cognitive function of aged rats after anesthesia

**DOI:** 10.1080/15685551.2022.2090678

**Published:** 2022-06-20

**Authors:** Hao-Lin Zhang, Qiang Jia, Fen Tian, Hui Zhang

**Affiliations:** a Department of Anesthesiology, Second Affiliated Hospital, Army Medical University, PLA, Chongqing, Chongqing, China; b Department of Pain, Hubei Provincial Hospital of Traditional Chinese Medicine, Wuhan, Hubei, China; c Department of Pain, Hubei Provincial Academy of Traditional Chinese Medicine, Wuhan, Hubei, China; d Department of Operating Room, Renmin Hospital of Wuhan University, Wuhan, Hubei, China; e Department of Anesthesiology, Hubei Provincial Hospital of Traditional Chinese Medicine, Wuhan, Hubei, China; f Department of Anesthesiology, Hubei Provincial Academy of Traditional Chinese Medicine, Wuhan, Hubei, China

**Keywords:** Coordination polymer, anesthesia, ELISA assay

## Abstract

Two Mn^2+^ coordination polymers (CPs) with the scientific terms of {[Mn(TTPA)·(H_2_O)_2_]·H_2_O}*_n_* (**1**) and {[Mn(TTPA)·(H_2_TTPA)]·2DMSO}*_n_* (**2**) were favorably created on the basis of multidentate linking organic ligand 2,5-bis-(1,2,4-triazol-1-yl)-terephthalic acid (H_2_TTPA) in the conditions of solvent-presupposed thermal reaction. To measure the influence of two Mn^2+^ coordination polymers with novel structures, the ELISA assay and real-time RT-PCR assay were conducted in this present research. First of all, the ELISA assay was conducted to measure the content of inflammatory cytokines released into the hippocampal tissue. In addition to this, the relative expression of the TAU protein in the brain was further determined with real-time RT-PCR assay.

## Introduction

Postoperative cognitive dysfunction (POCD) refers to a central nervous system (CNS) complication after anesthesia and surgery, and its clinical symptoms are memory loss, abstract thinking, disorientation, and social activity and fusion ability [1,2]. POCD is very common in elderly surgical patients, which not only affects the quality of life of patients, increases the medical burden, but also leads to an increase in the incidence of postoperative complications and the mortality rate of patients [3,4]. In recent years, a large number of studies have found that various pathogenesis of POCD ultimately work through a common pathway-neuroinflammation. Some scholars believe that neuroinflammation is the central link in the occurrence and development of POCD, and plays a key role in the occurrence and development of POCD.

Coordination polymers (CPs) have drawn much attention result from their diversified functions in luminescence, magnetism, gas-storage properties and biomedicine [5–7]. To promote the usefulness of CPs, various synthetic methods have been conducted, and numerous CPs with diversified structures and crystal stacking frameworks have been constructed. It has been proved that there exists a complex series of aspects affecting the structures of CPs, such as the structure of organic ligands, the types of the metal ions, the polarity of solvents the pH value, the reaction time, the reaction temperature, and so on [8–12]. Furthermore, diversified kinds of weak intermolecular interactions, such as H-bonding, π–π packing, metal–metal interactions and so on, are momentous in the course of self-assembly [13–16]. In addition, because of the variety of connecting patterns and high frame stability, polycarboxylate (benzene-tribenzoate, dicarboxylate and biphenyl tetra-car-box-yl-ate) and N heterocyclic (triazole, imidazole and their derivatives) ligands have been conducted widely as chelating or bridging connectors [17–20]. Currently, both of them are simultaneously utilized to form the functional CPs with special topological frameworks [21–23]. Based on the above considerations, two innovative Mn^2+^ CPs with the scientific terms of {[Mn(TTPA)·(H_2_O)_2_]·H_2_O}*_n_* (**1**) and {[Mn(TTPA)·(H_2_TTPA)]·2DMSO}*_n_* (**2**) were created on the basis of multidentate linking organic ligand 2,5-bis-(1,2,4-triazol-1-yl)-terephthalic acid (H_2_TTPA) in the conditions of solvent-presupposed thermal reaction. Single-crystal X-ray diffraction researches explain that complex **1** shows a 4,4-linked 2D-layered framework, which is established in a methanol–DMF–H_2_O integrated solution. Complex **2** presents a three-dimensional porous structure with a 4,6-dinodal three-dimensional structure with a void volume of 36 points 3% and is established in the condition of DMSO-methanol solvent (ratio is 3:1). In the biological section, the influence of compounds **1** and **2** on the cognitive function of aged rats after anesthesia was assessed by ELISA and real-time RT-PCR assay, the toxicity of the two compounds were evaluated at the same time.

## Experimental

### Chemicals and measurements

MnCl_2_ · 4H_2_O (AR, 99.9%) was acquired from Tianjin Guangfu Chemical reagent company, H_2_TTPA ligand (97%, AR) was obtained from Jinan Henghua Chemical reagent company, DMF, MeOH and DMSO was supplied by Shanghai Guoyao Chemical group company. IR spectra were collected via the FTIR-8400S spectrometer in the range of 500 to 4000 cm^−1^. EA were conducted by the Vario MACRO cube elemental analyzer. TGA was completed in the range of 25–800°C on a PE analyzer at a heating speed of 20°C per minute in the condition of N_2_ environment. X-ray powder diffractions were conducted via the Rigaku D/Max-2500 PC diffractometer with Mo-Kα radiation over the 2θ range of five to fifty degrees at normal temperature.

### *Preparation and characterization for {[Mn(TTPA)·(H_2_O)_2_]·H_2_O}*_n_ *(1) and {[Mn(TTPA)·(H_2_TTPA)]·2DMSO}*_n_ *(2)*

A mixture of 0.1 mmol MnCl_2_ · 4H_2_O, 0.05 mmol H_2_TTPA, 1 mL DMF,2 mL CH_3_OH, and 4 mL H_2_O was set in a 15 mL Parr PTFE lined oxidation-resisting steel container; next, the container was held at 130°C for 3 days. Next, the container was lowered to normal temperature at the speed of 1.5°C per hour. An achromatic rectangular bulk **1** was purified from the products and was deeper cleaned with distilled water and dehydrated in the open air. Compound **1** was formed in 54% yield on the basis of H_2_TTPA. Anal. calc. for C_12_H_14_MnN_6_O_8_: carbon is 33.90; hydrogen is 3.32, nitrogen is 19.76. Found (%): carbon is 33.67; hydrogen is 3.21, nitrogen is 19.86.

A mixture of 0.1 mmol MnCl_2_ · 4H_2_O, 0.05 mmol H_2_TTPA, 2 mL DMSO and 2 mL CH_3_OH was set in a 15 mL Parr PTFE lined oxidation-resisting steel container; next, the container was held at 130°C for 3 days. Next, the container was lowered to normal temperature at the speed of 1.5°C per hour. An achromatic bulk **2** was purified from the products and was deeper cleaned with distilled water and dehydrated in the open air. Compound **2** was formed in 41% yield on the basis of H_2_TTPA. Anal. calc. for C_28_H_26_MnN_12_O_10_S_2_: carbon is 41.54; hydrogen is 3.24, nitrogen is 20.76. Found (%): carbon is 41.58; hydrogen is 3.31, nitrogen is 20.26.

The X-ray figures were gathered by applying the SuperNova diffractometer. The intensity figures were measured via applying the CrysAlisPro program and switched format to the HKL documents. The SHELXS software based on direct method was applied to construct the primary structural models, and the SHELXL-2014 software based on the least-squares method was adjusted. All non-H atoms were specified anisotropically and the H atoms bridged to C atoms were produced in geometrical methods. Table 1 exhibits the crystal information of complexes **1** and **2**.

**Table 1.** Crystal information of complexes **1** and **2**

Identification code	1	2
Empirical formula	C_12_H_14_MnN_6_O_8_	C_24_H_14_MnN_12_O_8_
Formula weight	425.23	653.41
Temperature/K	296.15	296.15
Crystal system	triclinic	triclinic
Space group	P-1	P-1
a/Å	7.126(2)	7.256(2)
b/Å	7.8640(10)	10.4125(14)
c/Å	8.029(3)	12.5589(12)
α/°	65.136(4)	73.8710(10)
β/°	89.067(2)	84.962(5)
γ/°	70.0950(10)	79.872(3)
Volume/Å^3^	379.57(18)	896.6(3)
Z	1	1
ρ_calc_g/cm^3^	1.860	1.210
μ/mm^−1^	0.934	0.424
Data/restraints/parameters	1390/0/124	3704/30/212
Goodness-of-fit on F^2^	1.035	1.054
Final R indexes [I ≥ 2σ (I)]	R_1_ = 0.0795, ωR_2_ = 0.2146	R_1_ = 0.0433, ωR_2_ = 0.1132
Final R indexes [all data]	R_1_ = 0.0918, ωR_2_ = 0.2283	R_1_ = 0.0520, ωR_2_ = 0.1175
Largest diff. peak/hole/e Å^−3^	0.88/-1.71	0.76/-0.82
CCDC	2119627	2,119,628

### ELISA assay

The ELISA assay was implemented in the current study to estimate the inhibitory activation of compounds **1** and **2** on the releasing of inflammatory cytokines into the hippocampal tissue. This preformation was conducted strictly in accordance with the manufacturer’s instructions with some appropriate modifications. In short, 40 SD mice (6–8 weeks, 200–220 g) were used in the study. All the rats were kept at the standard status of 20–25°C, in 12 h light or night cycle. All the procedures in this measurement were allowed by the Animal Ethics Committee of China. The anesthesia surgery was performed on the animals. Next, compounds **1** and **2** were injected into the animal at the indicated concentrations. Finally, the content of inflammatory cytokines released into the hippocampal tissue was determined by ELISA assay. This research was conducted at least three times, and the results were presented as mean ± SD.

### Real time RT-PCR

To estimate the relative expression of the TAU protein in the brain after the compound treatment, real-time RT-PCR was implemented in the current study. This preformation was conducted totally followed the manufacturer’s instructions with some appropriate modifications. In a word, 40 SD mice (6–8 weeks, 200–220 g) were used in the study. All the rats were kept at the standard status of 20–25°C, in 12 h light or night cycle. All the procedures in this measurement were allowed by the Animal Ethics Committee of China. The anesthesia surgery was performed on the animals. Next, compounds **1** and **2** were injected into the animal at the indicated concentrations. After the indicated treatment, the hippocampal tissue was harvested and the whole RNA in the tissue was extracted by TRIZOL reagent. After measuring the quantity of the total RNA, which was then reversely transcripted into cDNA. In the end, the relative expression of the TAU protein in the brain was estimated by real-time RT-PCR, with the *gapdh* as the internal control. This research was conducted at least three times, and the results were presented as mean ± SD.

### CCK-8 assay

After the biological evaluation, the CCK-8 assay was conducted and the toxicity of the new compounds was further evaluated. This research was finished strictly in accordance with the manufacturers' protocols with only a little change. Briefly, the 293 T cells in the logical growth phage were collected and seeded into the 96 well plates at the final destiny of 5000 cells per well. After 12 hours incubation in an incubator, the new compounds were added into the wells for 48 hours indicated treatment. After that, the culture medium was discarded and new medium containing 10 μL CCK-8 reagent was added into the well. After 4 hours incubation, the absorbance of each well was measured at 450 mm. The viability of all the cells was calculated.

## Results and discussion

### Crystal structures

**Figure 1.**
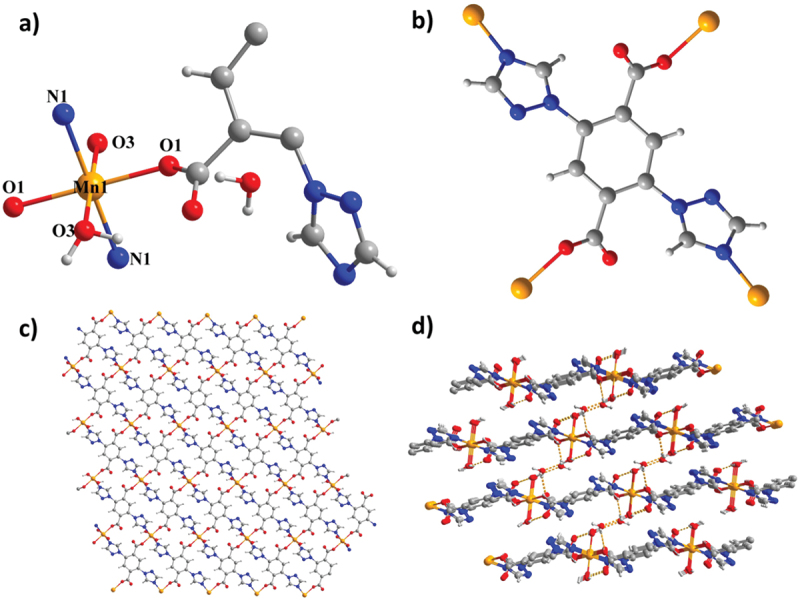
(a) Picture of the least building part of **1**. (b) View for the coordination mode of the ligand **1**. (c) The layered structure of **1**. (d) 3D packing diagram of **1** via the H-bond inter-reactions.

The single crystal X-ray detection demonstrates that complex **1** forms the crystal in the triclinic space group P-1, which displays a 2D-layered framework. Figure 1a shows that the least building part of **1** contained a single Mn ion with half space, a single half TTPA^2−^ ligand, a single coordinated H_2_O molecule and a single lattice H_2_O molecule. Figure 1a shows that the central Mn^2+^ exhibits a six-coordinated pattern and displays a usual octahedral framework, which is composed of double triazole nitrogen atoms from double TTPA^2−^ ligands, double μ_1_-η^1^ carboxyl oxygen atoms from the other double TTPA^2−^ anions and double H_2_O molecules. The Mn–O and Mn–N bond length range from 2.058(4) to 2.142(5). Moreover, every deprotonated TTPA^2−^ ligand is linked to four Mn^2+^ ions by double N atoms and double carboxylate groups, while every Mn^2+^ ion coordinates to four TTPA^2−^ ligands, which displayed as two types of 4-connection points (Figure 1b). Multidentate linking ligands link to the metals to construct a 2-D layer (Figure 1c). Unlimited and coordinated H_2_O molecules link to the neighboring layers into a 3-D supramolecular framework via H-bond inter-reactions (Figure 1d). The structure detection demonstrates that the coordination of solvent water promotes the construction of a 3-D structure. Thus, it may be probable to construct the 3-dimensional CPs structures under non-aqueous solvents.

**Figure 2.**
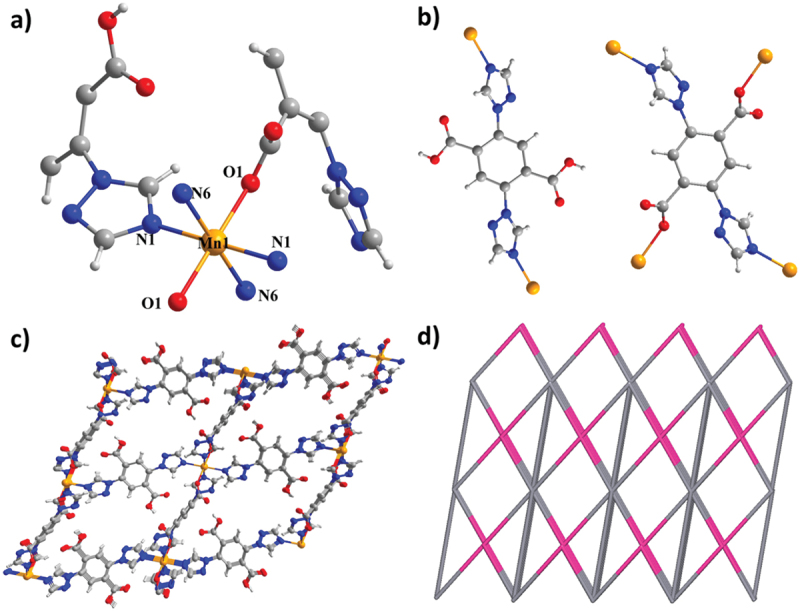
(a) Picture of the least building part of **2**. (b) View for the Ni8 cluster **2**. (c) The 3D-porous structure of **2**. (d) The (6,14)-connected 3D frame of **2**.

The single crystal X-ray detection demonstrates that complex **2** forms the crystal in the triclinic space group P-1, which displays a 3D-porous structure. Complex **2** contained a single Mn^2+^ ion, a single deprotonated TTPA^2−^ ligand, a single unprotonated H_2_TTPA molecule, and double lattice DMSO molecules in every asymmetric part (Figure 2a). The core Mn^2+^ ion displays the usual hexa-coordination pattern (MnO_2_N_4_) with four triazoyl nitrogen atoms from double TTPA^2−^ anions and double H_2_TTPA connectors and double carboxylate O atoms from the other double TTPA^2−^ linkers, which displays an octahedral structure. The completely deprotonated H_2_TTPA ligand is linked with four Mn^2+^ ions by double nitrogen atoms from the nitrogen triazole groups and double oxygen atoms from double μ1-η1 carboxylate groups, which may be seen as a planar linker (Figure 2b). The unprotonated H_2_TTPA displays a linear bridge connected with double Mn^2+^ ions by double triazolyl nitrogen atoms. The metal core was linked in harness via the planar linker and linear bridge, establishing a 3-dimensional framework with 1-D pathways following the *b* – xis (Figure 2c). From the aspect of topology, 4-connection planar ligands and 6-connection metal ions, along with the linear bridges are connected to others to construct a simplistic topology (Figure 2d). Pwt pattern proves that the pore volume is about 36 points 3%, which is occupied by unlimited DMSO molecules. The ratio of solvent molecules was estimated by elemental detection, single crystal X-ray diffraction and TG analysis.

**Figure 3.**
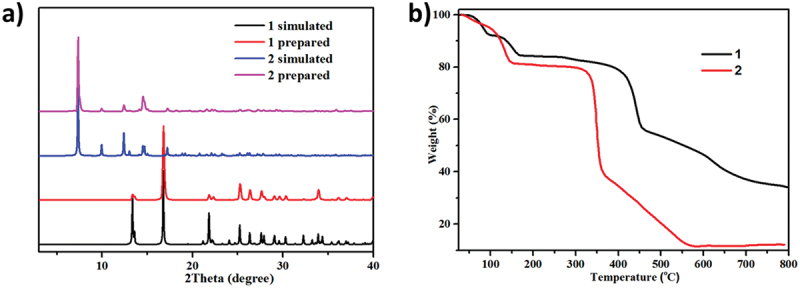
(a) The PXRD patterns for complexes **1** and **2**. (b) The TGA curves for complexes **1** and **2**.

To estimate the phase purity of the compounds, PXRD detections have been conducted for these compounds (Figure 3). The apex of the study and simulated PXRD pictures are in consistent with one another, illustrating that the crystal framework is authentically delegate of the blocky crystal compounds. The difference in strength may be due to the quality of the crystal sampled. The thermal steadiness of the three CPs was estimated by TGA in the nitrogen atmosphere and in the temperature ranging from 20°C to 800°C (Figure 3b). Complex **1** shows a weight loss of 16 point 7% in the range of 50–160°C, which is consistent with the evaporation of free and coordinated H_2_O molecules, which is identical to the theoretical value of 16 point 8%. When the temperature rose to 270°C, the framework began to decompose. As for complex **2**, it dispalys a weight loss of 19 point 6% (theoretical data: 19.43%) in the range of 40–160°C, which may be caused by the loss of unlimited DMSO molecules. The main structure can keep intact till 320°C.

### Compound significantly reduce the content of inflammatory cytokines released into the hippocampal tissue

**Figure 4.**
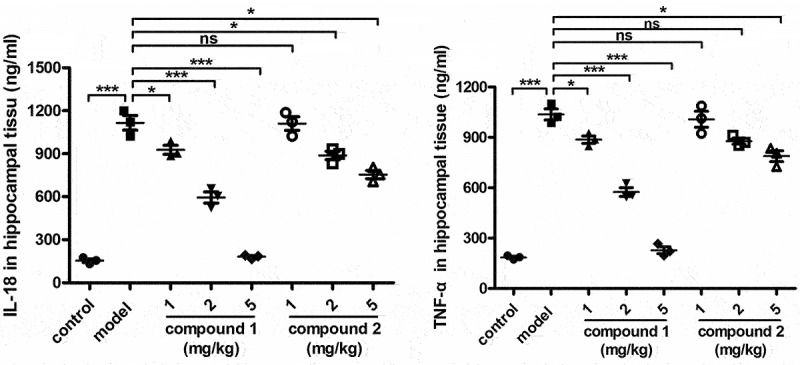
Significantly reduced content of the inflammatory cytokines in the hippocampal tissue after compound treatment. The anesthesia surgery was performed on the aged rats, then the compound was injected for treatment. The content of inflammatory cytokines released into the hippocampal tissue was determined by ELISA assay.

After the synthesis of compounds **1** and **2** with novel structures, their application values on improving the cognitive function of aged rats after anesthesia were evaluated. During the procession of cognitive function damage, there was usually combined with an increased level of inflammatory reaction in the hippocampal tissue. Therefore, the ELISA assay was first implemented and the content of inflammatory cytokines in the hippocampal tissue was estimated. Figure 4 reveals that the inflammatory cytokines released into the hippocampal tissue of the model group were higher than that of the control animal. However, after the treatment of compound **1**, the inflammatory response in the hippocampal tissue was obviously declined in a dose-dependent manner, which is much more excellent than that of compound **2**.

### Compound obviously inhibited the TAU protein relative expression in the brain

**Figure 5.**
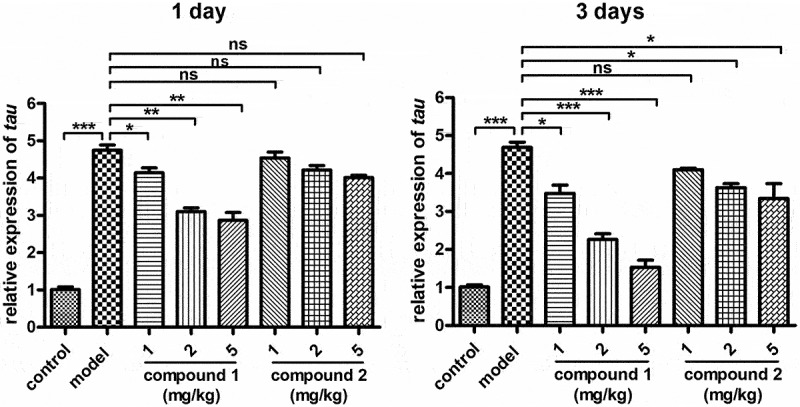
Obviously suppressed TAU protein relative expression in the brain after the compound. The anesthesia surgery was performed on the aged rats, then compounds **1** and **2** were injected at the indicated concentrations. The real-time RT-PCR was implemented and the relative expression of the TAU protein in the brain was measured.

In the above research, we have proved that compound **1** had a better biological effect than compound **2** on reducing content of the inflammatory cytokines in the hippocampal tissue. As reported, the expression of the TAU protein in the brain could be regulated by the inflammatory cytokines. Thus, the expression of the TAU protein in the brain was measured with real-time RT-PCR. The results in Figure 5 suggested that the level of TAU protein in the model group was much higher than the control group, which was significantly reduced by compound **1** in a dose-dependent manner. The biological activity of compound **2** was much weaker than compound **1**.

### Compound showed no toxicity on the 293 T cells

**Figure 6.**
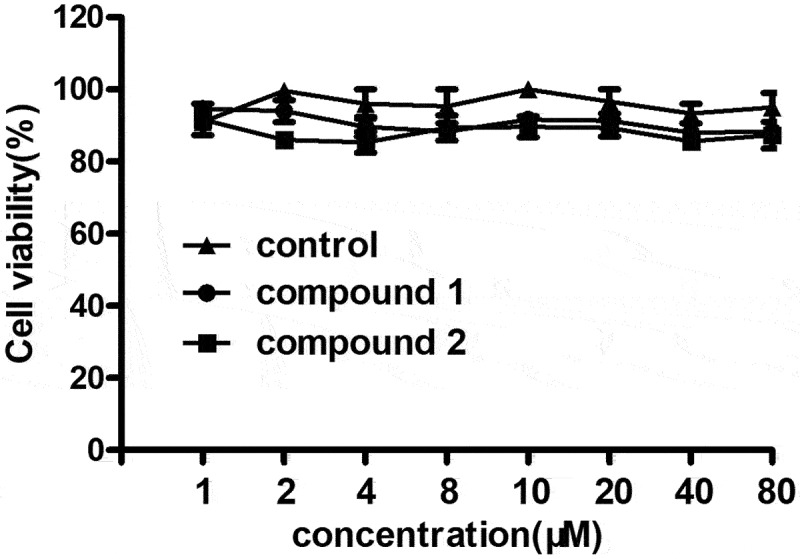
No toxicity of the new compound on 293 T cells. The 293 T cells were treated with compounds **1** and **2**, and the viability of the cells was measured with CCK-8 assay.

In the above research, we have proved that compound **1** was much better than compound **2** on the cognitive function improvement of aged rats after anesthesia. However, the toxicity of the new compound on 293 T still needs to be explored. So, the CCK-8 assay was conducted and the results are shown in Figure 6. We can see that compared with the control group, compounds **1** and **2** both showed no influence on the viability of the 293 T cells, suggesting the excellent application values of the new compound.

## Conclusion

In conclusion, we have created two Mn^2+^ CPs on the basis of multidentate bridging organic ligand 2,5-bis-(1,2,4-triazol-1-yl)-terephthalic acid (H_2_TTPA) in the conditions of solvent-presupposed thermal reaction. Single-crystal X-ray diffraction researches indicate that complex **1** shows a 4,4-linked 2D-layered framework, which is constructed in a methanol–DMF–H_2_O integrated solution. Complex **2** shows a 3-D porous structure with a 4,6-dinodal 3-D structure with a void volume of 36 points 3% and is formed in the conditions of volume of methanol–DMSO integrated solution (ratio is 3 : 1). The results of the ELISA assay demonstrated that compound **1** was more excellent than compound **2** on reducing the content of inflammatory cytokines in the hippocampal tissue. In addition to this, the relative expression of the TAU protein in the brain was also suppressed by compound **1**, but not compound **2** in a dose-dependent manner. Besides, compounds **1** and **2** both showed no influence on the viability of the 293 T cells, suggesting the excellent application values of the new compounds. Finally, we got this conclusion, compound **1** has a better application value than compound **2** on the promoting of cognitive function of aged rats after anesthesia through reducing the inflammatory response in the hippocampal tissue, as well as the relative expression of the TAU protein in the brain.
